# Identification of drug candidates targeting monocyte reprogramming in people living with HIV

**DOI:** 10.3389/fimmu.2023.1275136

**Published:** 2023-11-20

**Authors:** Rainer Knoll, Lorenzo Bonaguro, Jéssica C. dos Santos, Stefanie Warnat-Herresthal, Maartje C. P. Jacobs-Cleophas, Edda Blümel, Nico Reusch, Arik Horne, Miriam Herbert, Melanie Nuesch-Germano, Twan Otten, Wouter A. van der Heijden, Lisa van de Wijer, Alex K. Shalek, Kristian Händler, Matthias Becker, Marc D. Beyer, Mihai G. Netea, Leo A. B. Joosten, Andre J. A. M. van der Ven, Joachim L. Schultze, Anna C. Aschenbrenner

**Affiliations:** ^1^Systems Medicine, Deutsches Zentrum für Neurodegenerative Erkrankungen (DZNE), Bonn, Germany; ^2^Genomics and Immunoregulation, Life and Medical Sciences Institute, University of Bonn, Bonn, Germany; ^3^Department of Internal Medicine, Radboud University Medical Center, Nijmegen, Netherlands; ^4^Radboud Center for Infectious Diseases, Radboud University Medical Center, Nijmegen, Netherlands; ^5^Systems Hematology, Stem Cells & Precision Medicine, Max Delbrück Center - Berlin Institute for Medical Systems Biology (MDCBIMSB), Berlin, Germany; ^6^In Vivo Cell Biology of Infection, Max Planck Institute for Infection Biology (MPIIB), Berlin, Germany; ^7^Broad Institute at Massachusetts Institute of Technology (MIT) and Harvard, Boston, MA, United States; ^8^Ragon Institute of Mass General Hospital (MGH), MIT, and Harvard, Cambridge, MA, United States; ^9^Department of Chemistry, Institute for Medical Engineering and Science, Koch Institute, Cambridge, MA, United States; ^10^Platform for Single Cell Genomics and Epigenomics (PRECISE), DZNE and University of Bonn, Bonn, Germany; ^11^Institute for Human Genetics, University Hospital Schleswig-Holstein, Lübeck, Germany; ^12^Immunology and Metabolism, Life and Medical Sciences Institute, University of Bonn, Bonn, Germany; ^13^Department of Medical Genetics, Iuliu Hatieganu University of Medicine and Pharmacy, Cluj-Napoca, Romania

**Keywords:** systems immunology, transcriptomics, HIV, monocytes, inflammation, drug repurposing

## Abstract

**Introduction:**

People living with HIV (PLHIV) are characterized by functional reprogramming of innate immune cells even after long-term antiretroviral therapy (ART). In order to assess technical feasibility of omics technologies for application to larger cohorts, we compared multiple omics data layers.

**Methods:**

Bulk and single-cell transcriptomics, flow cytometry, proteomics, chromatin landscape analysis by ATAC-seq as well as *ex vivo* drug stimulation were performed in a small number of blood samples derived from PLHIV and healthy controls from the 200-HIV cohort study.

**Results:**

Single-cell RNA-seq analysis revealed that most immune cells in peripheral blood of PLHIV are altered in their transcriptomes and that a specific functional monocyte state previously described in acute HIV infection is still existing in PLHIV while other monocyte cell states are only occurring acute infection. Further, a reverse transcriptome approach on a rather small number of PLHIV was sufficient to identify drug candidates for reversing the transcriptional phenotype of monocytes in PLHIV.

**Discussion:**

These scientific findings and technological advancements for clinical application of single-cell transcriptomics form the basis for the larger 2000-HIV multicenter cohort study on PLHIV, for which a combination of bulk and single-cell transcriptomics will be included as the leading technology to determine disease endotypes in PLHIV and to predict disease trajectories and outcomes.

## Introduction

For people living with HIV (PLHIV), major risk factors for developing cardiovascular diseases (CVDs), neurocognitive impairment, frailty, and cancer are persistent low-grade inflammation and immune dysfunction even under long-term effective antiretroviral therapy (ART) (1–6). Although the adaptive immune system appears to play an important role (7), there is a growing body of evidence that suggests changes in the innate immune system as exemplified by elevated levels of circulating soluble CD163 and sCD14 derived from monocytes are critical (1, 8, 9). We and others have recently demonstrated that concentrations of pro-inflammatory monocyte-derived cytokines are elevated in serum from PLHIV, which was further validated when peripheral blood mononuclear cells were stimulated *ex vivo* with a number of pathogens or their derivatives resulting in increased levels of IL-1β (1, 10–14).

While CMV infection (15), the HIV reservoir itself (16), as well as microbial translocation (17) have been proposed as potential drivers of low-grade inflammation, the complex interplay between the different immune cell compartments in PLHIV is not fully understood. To study the role of different immune cells in the pathophysiology of persistent inflammation in PLHIV it will be necessary to apply higher-resolution single-cell technologies to larger cohorts of PLHIV (18–20). Based on our previous experience applying single-cell technologies to better understand the pathophysiology of COVID-19 (21–23) or chronic obstructive pulmonary disease (COPD) (24), we have recently suggested that large-scale studies should be preceded by smaller optimization studies for clinical application of omics technologies to a particular disease setting (25, 26).

Here, we describe a study using bulk and single-cell transcriptomics technologies as well as chromatin landscaping by ATAC-seq under clinically applicable conditions to assess the reprogramming of the peripheral immune cell compartment in PLHIV cohorts. Despite heterogeneity between individuals, scRNA-seq combined with bulk transcriptomics on a limited number of PLHIV included in this pilot study revealed important new information concerning the involvement of the monocyte compartment in persistent low-grade inflammation. Further, a reverse transcriptome approach in this setup allowed the identification of drug candidates reducing the inflammatory endophenotype, which we validated experimentally in an independent group of PLHIV.

## Results

### Bulk transcriptomes from PBMC of PLHIV are dominated by monocyte-related proinflammatory programs

**Figure 1:**
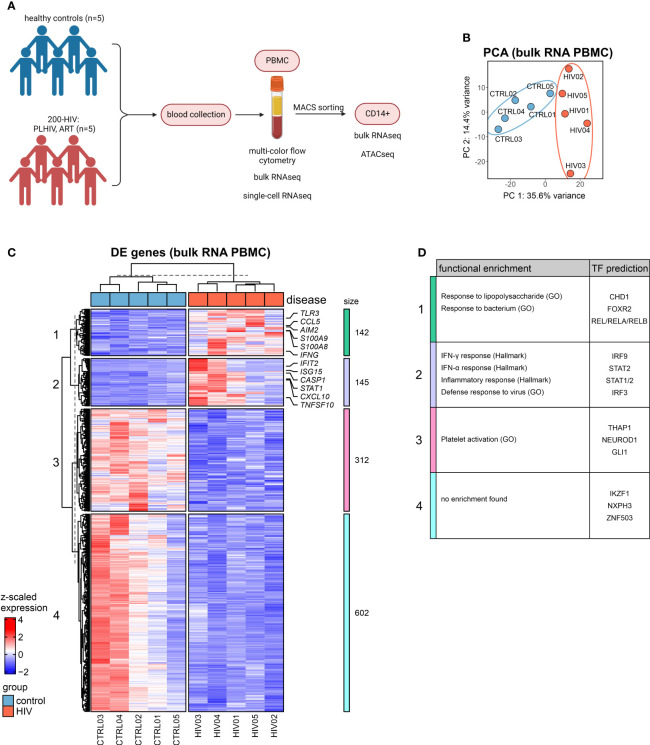
Bulk transcriptomes from PBMC in PLHIV are dominated by monocyte-related proinflammatory programs **(A)** Overview of the study design. **(B)** Principal component analysis (PCA) of bulk RNA-seq data from PBMCs. **(C)** Heatmap of DEG (adj. p.val<0.05, |FC|>1.5) from bulk PBMC transcriptomes based on HIV vs. control (see **Figure S1C**) and hierarchical clustering of genes into 4 clusters. **(D)** Functional enrichment using the GO and Hallmark databases and transcription factor (TF) prediction of gene clusters from **(C)** (full list see **Supplementary Table S2**).

We previously demonstrated in a cross-sectional study that PLHIV exhibits a proinflammatory profile in monocyte- but not lymphocyte-derived cytokines (1). We recalled five male PLHIV using long-term suppressive ART (mean 7.4 years) from the 200-HIV study with no overt clinical symptoms at the time of blood draw, determined as normal progressors, to investigate whether higher-resolution technologies down to the single-cell level would reveal further information about molecular and functional changes within the peripheral immune system in PLHIV. We generated a multi-layer dataset including selected soluble factors in plasma, multicolor flow cytometry (MCFC), bulk RNA-seq, Assay for Transposase-Accessible Chromatin using sequencing (ATAC-seq) and microwell-based scRNA-seq comparing five age- and sex-matched healthy controls (**Figure 1A**; **Supplementary Table S1**).

The MCFC data generated here indicate that the five PLHIV chosen were representative of the 200-HIV cohort with similar alterations in the circulating immune cell compartment (e.g. higher CD8+ and lower CD4+ T as well as NK cell population frequencies in PLHIV versus healthy donors) (11) (**Figure S1A**). Principal component analysis (PCA) of bulk RNA-seq of PBMC revealed a disease-associated separation of the samples (**Figure 1B**). Exploration of these alterations by differential gene expression analysis resulted in 287 up- and 914 down-regulated genes in PLHIV compared to control (|FC|>1.5, adj p-value <0.05, with independent hypothesis weighting (IHW) correction) (**Figure S1B**). Inspection of those differentially expressed genes (DEGs) in more detail by hierarchical clustering revealed four transcript clusters similarly regulated across the donors (**Figure 1C**). One cluster revealed a group of commonly upregulated early innate immune response genes for PLHIV and a second cluster contained typical interferon response genes (**Figure 1C**), which was corroborated by functional enrichment analysis (**Figure 1D**; **Supplementary Table S2**). Upregulation of alarmins *S100A8* and *S100A9* (cluster 1), which have been previously associated with inflammation (27, 28) indicated a strong signal from the myeloid cell compartment. In cluster 2, *STAT1*, previously linked to enhanced inflammation in HIV (29, 30), was strongly expressed. Both heatmap visualization (**Figure 1C**) and gene set variation analysis (**Figure S1C**) showed the highest heterogeneity among the five patients in genes belonging to cluster 2.

Collectively, analysis of bulk transcriptomes from PBMCs of PLHIV revealed upregulation of innate and myeloid proinflammatory gene programs.

### Bulk transcriptomics of monocytes in PLHIV reveals enriched IFN-signaling

**Figure 2:**
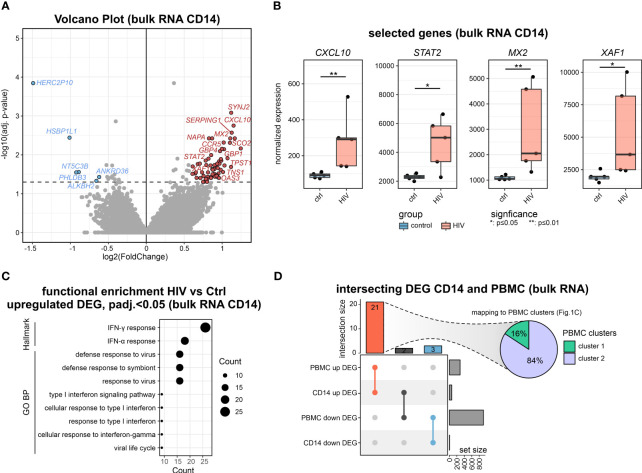
Bulk transcriptomics of monocytes in PLHIV mainly reveals IFN-signaling **(A)** Volcano plot showing the DEGs (adj. p.val<0.05, |FC|>1.5) in HIV vs. control of bulk CD14^+^ monocyte transcriptomes. **(B)** Boxplot and whisker of selected HIV-specific genes. Wilcoxon rank-sum for statistical testing (*: p-value <0.05, **: p-value <0.01). **(C)** Functional enrichment using the GO and Hallmark databases upregulated DEG (HIV vs Ctrl). **(D)** Intersecting DEG for the comparison of HIV vs Ctrl in bulk transcriptomes from CD14^+^ monocytes and PBMCs. Commonly upregulated DEG mapped to PBMC clusters from **Figure 1C**.

The bulk transcriptomes of PBMCs pointed towards the involvement of myeloid cells in PLHIV, and indeed plasma concentrations indicated elevated monocyte-specific soluble factors in circulation such as sCD163 and sCD14, a classical marker of HIV disease progression and monocyte activation (8, 31, 32), while other markers such as liver-derived C-reactive protein (CRP) did not show a significant elevation in these PLHIV (**Figure S2A**). Consequently, we isolated CD14^+^ monocytes from the same donors (**Figure S2B**) and analyzed their transcriptomes. DEGs were calculated for the comparison of PLHIV vs. control, resulting in 65 up- and 6 down-regulated genes (|FC|>1.5, p-value <0.05, IHW) (**Figures 2A**, **S2C**). Upregulated genes included several type I IFN-related genes such as *CXCL10*, *STAT2*, *MX2*, and *XAF1* (**Figures 2B**, **S2D**). Functional enrichment analysis of the upregulated DEGs supports these findings on the pathway level with IFN response and response to the virus being the most highly enriched terms (**Figure 2C**). The intersection of the CD14^+^ DEG with those from the PBMC data revealed 3 shared downregulated (*HERC2P10*, *HSBP1L1*, *PHLDB3*) and 21 upregulated (e.g. *CXCL10, SERPING1, GBP1*) genes, most of which belong to cluster 2 of the PBMC DEGs (**Figure 2D**).

To investigate a possible epigenetic component of the disease-associated changes, we performed ATAC-seq of sorted CD14^+^ monocytes. Using default analysis criteria (|FC|>1.5, adj. p-value < 0.05), we identified no differentially accessible regions (DARs) when comparing cells from PLHIV with control donors (**Figure S2E**).

Collectively, the CD14^+^ monocytes in PLHIV show clear signs of transcriptional activation of IFN-mediated pathways which is not significantly impacted by chromatin packing.

### “Anti-viral” monocyte state is persistent in PLHIV

**Figure 3:**
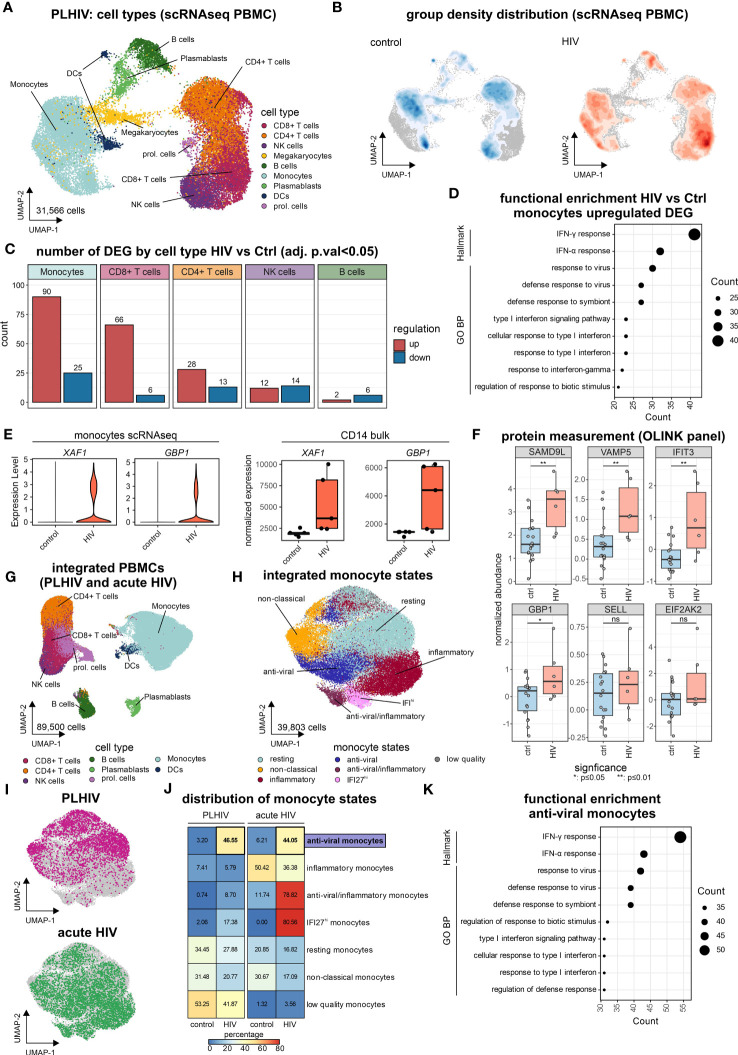
“Anti-viral” monocyte state is persistent in PLHIV **(A)** UMAP of PBMCs from PLHIV patients (n= 31,566 cells) indicating identified cell types. **(B)** UMAP from **(A)** colored by disease group density distribution. **(C)** Number of DEG (adj. p.val<0.05, |log2FC|>0.25, min.pct=0.1) by major cell types for the comparison HIV vs Ctrl. **(D)** Functional enrichment using the GO and Hallmark databases for HIV-specific (up-regulated) genes in monocytes. **(E)** Marker expression of *XAF1* and *GBP1* by disease group for monocytes extracted from scRNA-seq data (left panel) and bulk CD14^+^ monocytes (right panel). **(F)** Protein level quantification for SAMD9L, VAMP5, IFIT3, GBP1, SELL, and EIF2AK2 using the Olink system. Wilcoxon rank-sum for statistical testing (ns: not significant, *: p-value <0.05, **: p-value <0.01). **(G)** UMAP of integrated PBMCs from PLHIV **(A)** and acute HIV (Kazer et al., n= 59,286 cells) for commonly present cell types in both datasets, identified cell types are indicated (total dataset n= 89,500 cells). **(H)** UMAP of integrated monocyte subset (n= 39,803 cells) from PLHIV and acute HIV annotated by signatures from Kazer et al. and cluster marker expression. **(I)** UMAP of integrated monocytes colored by dataset origin (PLHIV and acute HIV), each n= 10,000 cells. **(J)** Confusion matrix heatmap showing the distribution of monocyte cell states for disease groups stratified by dataset. **(K)** Functional enrichment using the GO and Hallmark databases for markers (from **Figure S3E**) of the ‘anti-viral’ monocyte state.

To address whether changes in the transcriptomes of PBMCs (**Figure 1**), as well as isolated CD14+ monocytes (**Figure 2**), are due to general alterations in the transcriptional programs of the myeloid compartment or due to the presence of disease-specific cell states, scRNA-seq was performed on PBMCs of the same individuals (**Figure 3**). Transcriptomes from 31,566 single cells were produced representing all major immune cell types of the peripheral circulation according to cluster-specific markers known in literature, such as monocytes (*LYZ, S100A9, S100A8*), CD4^+^ T cells (*IL7R, TRAT1*), CD8^+^ T cells (*GZMH, CCL5, CD3G*) and NK cells (*GNLY, NKG7, KLRF1*) (**Figures 3A**; **S3A**). Density-based coloring of the UMAP for PLHIV and control groups disclosed a major transcriptional shift in the monocyte cluster, in the CD8^+^ T cell cluster, but not in the CD4^+^ T cell cluster (**Figure 3B**). These differences are also reflected in changes in the number of DEG (log2FC=0.25, adj. p-value<0.05, min.pct=0.1) (**Figure 3C**). Compared to other immune cell populations, monocytes showed the highest number of DEGs comparing PLHIV with controls, 90 up- and 25 down-regulated genes. Functional enrichment analysis on the HIV-specific up-regulated DEG of the monocyte compartment included terms such as ‘IFN-γ response”, “IFN-α response” and “response to virus” (**Figure 3D**), in line with the PBMC and CD14 bulk RNA-seq data (**Figures 1B**, **2C**). Similar to the bulk data produced from CD14^+^ monocytes, 19 genes were also upregulated in the monocyte cluster resulting from scRNA-seq, including *XAF1* and *GBP1* (**Figures S3B, E**; **Supplementary Table S3**). To confirm the upregulation of the genes from that intersection, we measured protein levels of SAMD9L, VAMP5, IFIT3, GBP1, SELL, and EIF2AK2, which are all related to IFN responses (**Figure 3F**). In PLHIV, all six proteins showed elevated levels compared to healthy controls with SAMD9L, VAMP5, IFIT3, and GBP1 being significant.

To relate our findings from chronic HIV in PLHIV to acute HIV, in which patients did not yet receive ART and have high viremia, we integrated the newly produced data with our previously published results using the same microwell-based single-cell technology describing several inflammatory monocyte states in acute HIV infection (33) resulting in 89,500 single-cell transcriptomes (**Figures 3G**, **S3C, D**). To investigate the possible presence of chronic disease-specific cell states within the monocyte compartment, we subsetted the monocytes of the integrated scRNA-seq dataset (**Figure 3H**). Clustering of the monocyte compartment resulted in seven monocyte substrates, which could be annotated based on the previously reported acute monocyte states (33). These included several inflammatory monocyte states associated with acute HIV infection, e.g. anti-viral/inflammatory or *IFI27*^hi^ monocytes (**Figures 3H**, **S3E**). Monocytes from our new data predominantly exhibited resting and non-classical states, irrespective of HIV group (**Figures 3I, J**). Chronic HIV was characterized by an ‘anti-viral’ monocyte state that was also found during acute infection (**Figure 3J**). This ‘anti-viral’ monocyte state expresses interferon-related genes, e.g. *IFIT3* and *ISG15* (**Figure S3E**), and is strongly enriched for the hallmarks ‘IFNγ response’ and ‘IFNα response’ as well as the GO term ‘response to virus’ (**Figure 3K**), reminiscent of our results in PBMCs (**Figures 1C, D**) and CD14^+^ monocytes (**Figure 2C**).

Even within the resting and non-classical monocyte substates that do not exhibit major changes in proportions between the clinical groups (**Figure 3J**), differentially expressed genes (log2FC=0.25, adj. p-value<0.05, min.pct=0.1) for PLHIV vs. controls (resting: 70 DEGs, non-classical: 36 DEGs) had a substantial overlay with the DE genes identified from bulk PBMC data, i.e. clusters 1 and 2 (**Figures 1C**, **S3F**; **Supplementary Table S3**). ScRNA-seq also revealed heterogeneity in cell state distribution in the group of the PLHIV, which was not apparent in the healthy individuals (**Figure S3G**).

Collectively, single-cell transcriptomics identified monocytes as the major cause of changes in PLHIV. Common alterations were evident across all identified cell states, including resting and non-classical monocytes, yet scRNA-seq uncovered elevated numbers of monocytes in the ‘anti-viral’ cell state in chronic HIV that had been described for acute HIV infection. Thus, pathology in PLHIV is a combination of molecular alterations and proportion changes that could only be revealed by analysis on the single-cell level.

### Drug repurposing to reverse monocyte reprogramming in PLHIV

**Figure 4:**
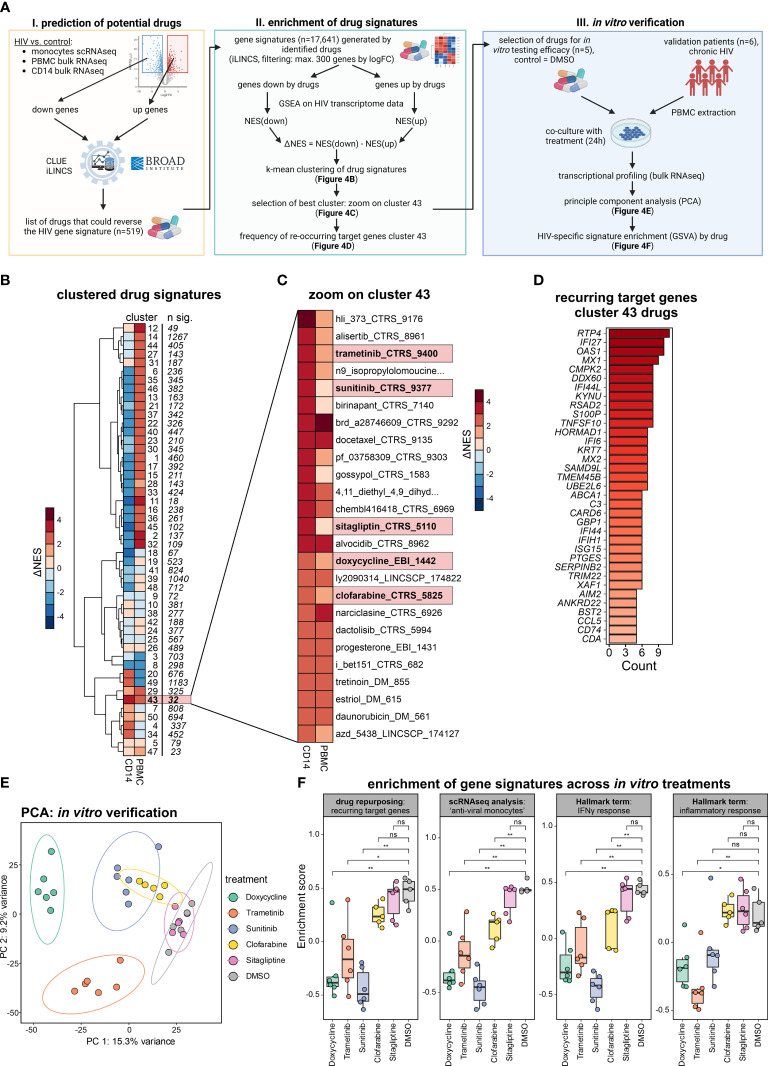
Drug repurposing to reverse monocyte reprogramming in PLHIV. **(A)** Drug prediction workflow and follow-up *in vitro* verification, NES=normalized enrichment score. **(B)** Heatmap showing hierarchical clustering (k-mean=50) of ΔNES from all drug signatures (n= 17,641) as groups enriched on transcriptomes from bulk CD14^+^ monocytes and bulk PBMCs. **(C)** Zoom into cluster 43 from **(B)**, depicting all involved drug signatures. **(D)** Recurring target genes of drug signatures identified in cluster 43 from **(C)**. **(E)** Principal component analysis (PCA) of bulk PBMC transcriptomes of the *in vitro* verification experiment (five HIV donors with six conditions). Samples colored by treatment, DMSO as untreated control. **(F)** Enrichment of gene signatures across *in vitro* treatments, analyzed signatures include the recurring target genes from cluster 43 (drug repurposing), marker for the ‘anti-viral’ monocytes (integrated scRNA-seq analysis), and the hallmark terms ‘IFNγ response’ and ‘inflammatory response’. Wilcoxon rank-sum for statistical testing (ns: not significant, *: p-value <0.05, **: p-value <0.01).

To illustrate how to identify potential drug targets for reversing a molecular phenotype, here the changes observed in monocytes, we performed a drug repurposing approach (**Figure 4A**) following a previously established methodology (34). In brief, genes up- and down-regulated in PLHIV who are under ART from scRNA-seq monocytes, bulk RNA-seq PBMCs, and bulk RNA-seq CD14^+^ monocytes were subjected to the drug prediction databases iLINCS and CLUE (35, 36), resulting in 519 predicted drugs (**Supplementary Table S4**). From those drugs, 17,641 signatures were retrieved from iLINCS and used as input for GSEA on the bulk RNA-seq CD14^+^ monocytes and PBMC datasets. Drug signatures were then clustered by their delta normalized enrichment score (ΔNES), resulting in 50 clusters (**Figure 4B**; **Supplementary Table S4**). The ΔNES indicates the efficiency of the respective drug signature to reverse the PLHIV-specific signature, with higher ΔNES indicating a more complete reversal. Cluster 43, consisting of 32 signatures, showed the highest ΔNES for CD14^+^ monocytes and also a high ΔNES for PBMCs (**Figure 4C**). To decipher the commonalities of those drug responses, we investigated recurring target genes of all drug signatures in the cluster (**Figure 4D**). A majority of genes were interferon-associated such as *IFI27*, *OAS1*, *MX1*, and *IFI44L*, and the target genes were strongly enriched in the ‘anti-viral’ and ‘anti-viral/inflammatory’ monocyte states (**Figure S4A**).

Of the 32 drug signatures, we chose five among the top 20 drugs according to ΔNES for CD14^+^ monocytes in PLHIV. Four of them had been studied in the context of HIV infection [trametinib (37), sunitinib (38, 39), sitagliptin (40, 41), clofarabine (42)], but had not been reported to alter transcriptional programs in monocytes. Additionally, the predicted antibiotic doxycycline, for which neither anti-viral nor immune-modulating function has been reported, was chosen as well. Instead of addressing the viral life cycle, this approach predicts a potential impact on the host’s immune response to these drugs. To test this hypothesis and validate our predictions, we performed *in vitro* experiments stimulating PBMC from PLHIV with the respective drugs.

Six independent PLHIV were recruited, PBMCs were isolated and co-cultured in the presence of the selected drugs or with DMSO as control (**Figure 4A**, right panel). After overnight incubation, RNA was extracted and bulk transcriptomics was performed to measure transcriptional changes induced by the respective treatment (**Figure S4B**). The different *in vitro* treatments resulted in prominent transcriptional changes in the PBMCs, evident in the PCA with the strongest alterations induced by doxycycline followed by trametinib, sunitinib, and clofarabine (**Figure 4E**). Differential expression analysis reflected this finding in the number of DE genes (**Figure S4C**). Of note, doxycycline, trametinib, and sunitinib induced a greater number of downregulated DEGs.

Based on our previous findings, we tested the influence of the different treatments by analyzing the reduction of gene signature enrichment for 1) the recurring target genes of cluster 43 identified from the drug repurposing pipeline (n=35), 2) the ‘anti-viral monocyte’ markers from our integrated single-cell RNA-seq analysis (n=137), and the hallmark terms 3) ‘IFNγ response’ (n=200) and 4) ‘inflammatory response’ (n=200) (**Figure 4F**). Sunitinib and doxycycline showed the most significant impact, strongly reversing the four different HIV-specific gene signatures. Trametinib also showed strong, clofarabine a moderate, and sitagliptin no reductions of the four signatures in our *in vitro* verification experiment. These differential effects of the different drugs are also seen on the gene level when investigating the top leading edge genes of the four signatures by each drug (**Figure S4D**).

Taken together, we predicted drugs that could reverse the altered monocyte-derived signatures and confirmed our repurposing approach *in vitro* with the drugs doxycycline and sunitinib strongly reversing the HIV-specific gene signatures, making them repurposed drug candidates of interest.

## Discussion

In the present study, we illustrate in a small group of PLHIV derived from our previous cross-sectional 200-HIV cohort study (1) that single-cell and bulk transcriptomes of isolated immune cells revealed reprogramming in multiple cellular compartments in PLHIV, with innate immune cells, in particular monocytes, showing most profound changes. We further illustrate that a certain cellular state of monocytes, previously reported in acute HIV infection can be observed in PLHIV, while other cell states associated with acute inflammation are specific for acute HIV and absent in PLHIV. Long-term usage of ART in PLHIV results in undetectable viral loads and restores CD4 cell counts to normal levels, and therefore PLHIV patients differ from people with an acute HIV infection that have high-level viremia and reduced CD4 cell counts (33). Despite the small number of PLHIV studied, which clearly showed heterogeneity in their transcriptional profiles, we also illustrate that combined bulk and single-cell data of these PLHIV was already sufficient to predict drug candidates for reversing the observed transcriptional deviations in the monocyte compartment. While technically applicable to a cohort study setting, ATAC-seq of this small number of PLHIV did not reveal any significant differences, which clearly points towards the need for larger cohorts when assessing chromatin landscape differences. As such the study reported here provides the necessary information to include sophisticated transcriptome and epigenome data generation to be integrated into the larger 2000-HIV cohort study currently recruiting PLHIV including elite controllers.

The combined analysis of bulk transcriptomes from PBMC and purified CD14^+^ monocytes together with single-cell transcriptomes from blood allowed us already in a rather small number of PLHIV to define major changes within the peripheral immune cell compartment, e.g. the identification of a gene cluster characterized by IFN signaling. The higher-resolution information from scRNA-seq revealed that some of the changes observed in the PBMC-derived transcriptomes was due to molecular changes in monocytes including cell-state differences, but not due to cell-type distribution differences, further supporting the use of higher-resolution technologies in larger cohort studies. While IFN-signaling related molecular changes (cluster 2, **Figures 1C, D**) were also captured in bulk transcriptomes from purified CD14^+^ monocytes (**Figure 2**), the overall information content from purified CD14^+^ monocytes was surprisingly low, indicating that many of the changes observed in PBMC are derived from other monocyte cell states (CD14^low/-^) and other cell types. Single-cell transcriptomes clearly corroborated this hypothesis showing that basically all immune cell types exhibited transcriptional changes in PLHIV. With the lowest information content and highest technical effort, we concluded that cell-type isolation procedures are not suitable for larger cohort studies on PLHIV. Moreover, when assessing DEG in monocytes using both bulk and single-cell transcriptomes, we detected less DEG in bulk and only a small intersection with single-cell data (n=19, **Figure S3B**). Differences in experimental sample handling or sequencing resolution could explain this small intersection, however, even though certain genes were not tested to be significantly altered in both methods, the general pathway activation towards IFN responses was uncovered by both methods.

The systemic assessment of single-cell transcriptomes derived from PBMC of PLHIV revealed that major transcriptional reprogramming was mainly observed in monocytes and CD8^+^ T cells with fewer changes in CD4^+^ T cells, NK cells, and B cells. Focusing on the cell compartment with the major changes, we revealed a cell state composition in PLHIV including the well-described classical and non-classical monocyte states, but also a cell state we previously termed ‘anti-viral monocytes’ in acute HIV infection (33). Interestingly, this particular cell state showed high heterogeneity between PLHIV, which will have to be studied in larger cohorts to better define whether there is a pattern reminiscent of PLHIV endotypes or whether this might be explained by individual clinical incidents prior to blood sampling. Despite the heterogeneity of this monocyte state, the assessment of genes enriched in gene clusters derived from bulk transcriptomes indicated that even classical and non-classical monocyte states in PLHIV are characterized by elevated expression of cluster 2 genes, supporting the notion that despite the observed heterogeneity, persistent IFN signaling seems to be a major hallmark of persistent inflammation in PLHIV (43). Based on these informative and promising results we propose to integrate these levels of omics technologies into larger PLHIV studies.

As we identified a major theme for persistent inflammation in this small number of PLHIV, we addressed whether this information would already be sufficient to identify drug candidates by a reverse transcriptome approach (34). Interestingly, while most therapeutic strategies are currently addressing alternative antiviral drugs with less toxicity or treatment strategies aiming at minimizing ART toxicity, fewer drug regimens address immunomodulation itself including the use of purinergic P2X receptor inhibitors (44) or statins (45–49). In clinical studies testing the efficacy of these therapeutic approaches to lower inflammation in PLHIV, mainly soluble mediators (e.g. CRP, sCD14, IL-6, sCD163) measured in plasma or serum were used as readouts, while high-resolution technologies to address molecular changes in immune cells were not reported. We exemplified here, how such an approach could be applied to the identification of drug candidates lowering the inflammatory response observed in PLHIV. We focused on a cluster of drugs with a particularly high probability of reversing the transcriptional alterations observed in monocytes and experimentally validated a small number of drug candidates. A surprising finding was that the antibiotic doxycycline induced the strongest effect mainly reducing gene expression. Together with sunitinib, doxycycline was most effective in reversing gene expression alterations of 1) the major target genes used for drug prediction, 2) of the marker genes expressed in monocytes with the ‘antiviral’ cell state, 3) of the hallmark genes related to IFN signaling, and 4) hallmark genes related to the pro-inflammatory response. These findings strongly suggested that drugs such as doxycycline might not only function as antibiotics but also modulate host immune responses. This is similarly true for the drug candidates sunitinib and trametinib, which have been developed for completely different purposes (50, 51). Importantly, the modulation of the monocyte-related immune activation should not be considered yet as being unrestrictedly helpful for PLHIV, as it is not yet entirely clear whether these gene programs would play a clinically beneficial role or not. As these findings have to be considered as proof-of-concept, further investigations using more drug candidates, different drug concentrations, and further optimized computational and miniaturized experimental procedures in a larger group of PLHIV are certainly warranted to more quickly identify promising new drug candidates counteracting the inflammatory state in PLHIV under ART therapy.

### Limitations of the study

The present study was conceptualized based on the previous cross-sectional 200-HIV cohort study (1) to determine whether the combination of high-resolution and high-content technologies such as bulk and scRNA-seq data would lead to additional insights into the pathophysiology of immune deviations in PLHI and therefore, only a limited number of individuals were included in this study. As the main purpose was to determine the best strategy to scale these technologies to larger clinical cohorts, we were surprised that despite a rather small number of individuals studied and obvious heterogeneity within the group of PLHIV, we could retrieve important information about major molecular changes on transcriptome level in all immune compartments. However, it became also clear that other layers, e.g. chromatin landscapes as assessed by ATAC-seq require a much larger number of individuals to determine whether immune cells in PLHIV are also altered on this epigenetic level. Based on these initial findings, we have now started to include these technologies in the much larger 2000-HIV cohort study of approx. 2000 PLHIV to study aspects such as disease heterogeneity, potential disease endotypes, and association of cellular changes with clinical trajectories, or to determine potential biomarkers predicting disease outcome. Certainly, the observation that innate immune cells such as monocytes show the most pronounced transcriptional reprogramming in PLHIV was unexpected and will be one major focus within the currently being assembled cohort of PLHIV. Moreover, the identification of these monocyte-derived programs also opens new avenues toward the identification of new mechanisms on how transcriptional alterations contribute to immune dysregulation in PLHIV.

## Methods

### Lead contact

Dr. Anna C. Aschenbrenner, anna.aschenbrenner@dzne.de.

### Materials availability

This study did not generate unique reagents.

### Data and code availability

Bulk RNA-seq datasets and single-cell RNA-seq data have been deposited at the European Genome-phenome Archive (EGA) and are publicly available under the accession numbers.

All original code is stored on FASTGenomics: https://beta.fastgenomics.org/p/HIV_Pilot

Any additional information required to reanalyze the data reported in this paper is available from the lead contact upon request.

### Study cohort

Five PLHIV were recruited from the outpatient HIV clinic of the Radboud University Medical Center on March 26-28th 2019. Included patients were five males of Dutch/Western-European ethnicity who were receiving cART for more than 6 months and latest HIV-RNA levels ≤200 copies/ml. Ethical approval was granted by the Ethical Committee of the Radboud University Medical Center Nijmegen, the Netherlands under registration number NL42561.091.12). Additionally, five age-/sex-matched healthy volunteers were included as controls (age 43-61), and ethical approval was granted by the Ethical Committee of the Radboud University Medical Center Nijmegen, the Netherlands under registration number NL32357.091.10). For the *in vitro* verification experiments of drugs, six additional male PLHIV were recruited (age 26-43, with ethical approval granted by the Ethical Committee of the Radboud University Medical Center Nijmegen, the Netherlands under registration number NL68056.091.18). Written consent was obtained from all participants involved in this study and experiments were conducted according to the Declaration of Helsinki principles.

### PBMC isolation

Human peripheral blood mononuclear cells (PBMCs) were isolated by dilution of blood in pyrogen-free PBS and differential density centrifugation over Ficoll-Paque (GE Healthcare, UK) as previously described by (52). Briefly, the interphase layer was collected, and cells were washed with cold PBS. Cells were resuspended in RPMI 1640 culture medium (Roswell Park Memorial Institute medium; Invitrogen, USA) supplemented with 50 g/mL gentamicin, 2 mM glutamax (Gibco, Life Technologies, USA), and 1 mM pyruvate (Gibco) and quantified. A fraction of PBMCs was viably frozen for later use. The cell suspension was spun down for 5 min at 300g, 4°C, after which all supernatant was removed. Cells were very gently resuspended in freezing medium (90% fetal calf serum, 10% DMSO) and aliquoted into cryovials. They were placed first at -80°C in a CoolCell freezing container (Corning), after which they were transported the next day on dry ice and moved to liquid nitrogen storage. For the thawing of PBMCs, one vial of 5 million cells was thawed in 10ml RMPI medium supplemented with 10% FCS.

### Preparation of Seq-Well arrays/libraries/sequencing

Seq-Well arrays and libraries were prepared from isolated PBMCs as described previously (24).

### Measurements of plasma markers

Clinical plasma markers were measured using ELISA (Duoset or Quantikine, R&D Systems) for IL18-BP, IL-18, hsCRP, sCD14, sCD163 or using SimplePlex Cartridges (Protein Simple) for IL-6, all performed according to manufacturers’ instructions. As a reference, the mean of healthy controls from van der Heijden et al. (1) were used.

### Isolation of CD14+ monocytes

CD14+ monocytes were isolated from PBMC by magnetic-activated cell sorting (MACS) positive selection with CD14 microbeads (Miltenyi Biotec), according to the manufacturer’s instructions. Depending on the available PBMC counts used as input, either MS or LS columns were used (Miltenyi Biotec). After isolation, cells were again resuspended in a Dutch modified RPMI culture medium (Invitrogen) supplemented with 50 µg/mL gentamycin, 2 mM glutamax and 1 mM pyruvate (Gibco, Life Technologies).

### Flow cytometry

Frozen PBMCs were thawed then stained for surface markers (**Supplementary Table S1**) in DPBS with BD Horizon Brilliant Stain Buffer (Becton Dickinson) for 30min at 4°C. To distinguish live from dead cells, the cells were incubated with LIVE/DEAD Fixable Yellow Dead Cell Stain Kit (1:1000 – Thermo Scientific). Following staining and washing, the cell suspension was fixed with 4% PFA for 10 min at room temperature to prevent any possible risk of contamination due to aerosol formation during sample handling and acquisition. Flow cytometry analysis was performed on a BD Symphony instrument (Becton Dickinson) configured with 5 lasers (UV, violet, blue, yellow-green, red).

### ATAC-seq

Frozen PBMCs were thawed and sorted on a BD FACSAria III (Blue, Yellow-Green, Red, and Violet lasers), and 20,000 live CD14^+^ cells were sorted and spun down at 500×g for 5 min at 4°C. The cell pellet was washed with 50 μL of cold 1x PBS buffer and spun down at 500 ×g for 5 min at 4°C. The pellet was then resuspended in 50 μL of cold lysis buffer (10 mM Tris-HCl, pH 7.4, 10 mM NaCl, 3 mM MgCl2, 0.1% IGEPAL CA-630) and spun down immediately at 500×g for 10 min at 4°C. The supernatant was then discarded, and the transposition reaction was immediately performed. To perform the transposition reaction, a mixture of transposase, 5x TAPS-DMF buffer (50mM TAPS (T5130 SIGMA), 25mM MgCl2, 50% DMF (N,N-Dimethylformamide)), and water was combined and added to the cell pellet. The transposition reaction was incubated at 37°C for 30 min. Following transposition, the DNA was purified using a Qiagen MinElute Kit. The transposed DNA was eluted in 10 μL of water, and purified DNA was stored at 4°C until the following day or at -20°C.

To amplify the transposed DNA fragments, a PCR mixture was prepared using the purified DNA, nuclease-free water, customized Nextera PCR primers, and NEBNext High-Fidelity 2x PCR Master Mix. The PCR mixture was cycled as follows: 72°C for 5 min, 98°C for 30 sec, 98°C for 10 sec, 63°C for 30 sec, and 72°C for 1 min. Steps 3-5 were then repeated 11 times for a total of 12 cycles. The PCR products were then purified using a Qiagen MinElute Kit and eluted in 12 μL of water. To validate the quality and concentration of the PCR products, gel electrophoresis was performed using the TapeStation and Agilent High Sensitivity D1000 kit.

### Protein measurements

Proteomic profiling of selected markers was performed as described before (53). In brief, venous whole-blood samples were collected in EDTA tubes and centrifuged into plasma, and then stored at -80°C. Protein measurements were performed by Olink Proteomics AB using the Olink Explore platform. QC and normalization were performed by Olink services. For this study, protein markers of interest were selected.

### *In vitro* verification of selected drugs

To verify the effectiveness of predicted drugs, six different PLHIV from the 200-HIV cohort were re-called, and the PBMCs were extracted and seeded in triplicates with 500,000 cells per replicate. The PBMCs were cultured for 24 hours in the presence of a selected subset of drugs from cluster 43, including trametinib (50 mM in 0.000002% DMSO), sunitinib (100 mM in 0.0001% DMSO), clofarabine (100 mM in 0.00001% DMSO), doxycycline (100 mM in H_2_O) and sitagliptin (100 mM in 0.0001% DMSO) or DMSO (0.001%) as control. After incubation, replicates were collected in a total of 1 ml TRIzol reagent and processed for bulk RNA-seq.

### Quantification and statistical analysis

#### RNA-sequencing analysis (bulk RNA PBMC, CD14)

Sequenced reads were aligned and quantified using STAR: ultrafast universal RNA-seq aligner (v2.7.3a) (54) and the human reference genome, GRCh38p13, from the Genome Reference Consortium. Raw counts were imported using the DESeqDataSetFromMatrix function from DESeq2 (v1.32.0) (55) and rlog transformed according to the DESeq2 pipeline. DESeq2 was used for the calculation of normalized counts for each transcript using default parameters. All normalized transcripts with a maximum overall row mean lower than 10 were excluded resulting in 26,920 present transcripts. All present transcripts were used as input for principal component analysis (PCA). Differentially expressed genes were calculated for HIV vs. control using an independent hypothesis weighting (IHW) adjusted p-value cutoff of 0.05 and an absolute fold change (|FC|) of 1.5. DEGs were used as input for the k-mean clustered heatmap (k=4), generating four clusters.

#### RNA-sequencing analysis (drug verification analysis)

Sequenced reads were aligned and quantified using kallisto v0.44.0 (56) and the human reference genome, GRCh38p13, from the Genome Reference Consortium. Raw counts were imported using the DESeqDataSetFromTximport function from DESeq2 (v1.32.0) (55) and vst-transformed according to the DESeq2 pipeline. DESeq2 was used for the calculation of normalized counts for each transcript using default parameters. All normalized transcripts with a maximum overall row mean lower than 10 were excluded resulting in 37,952 present transcripts. Variation in the data was identified using the SVA package (v3.40) (57), and batch effects were removed with limma (v3.48.3) (58) using the first six surrogate variables (SVs), which were also added in the design of the dds object. All present transcripts were used as input for principal component analysis (PCA) of the batch-corrected data. Differentially expressed genes were calculated for HIV vs. control using a p-value cutoff of 0.05, an adjusted p-value (IHW) < 0.05 (independent hypothesis weighting), and a |FC|>2. DEGs were used as input for the clustered heatmap.

#### Transcription factor prediction analysis

The R package RcisTarget (version 1.12.0) (59) was used to predict the transcription factors potentially regulating heatmap cluster-specifically contained gene sets. The genomic regions of TF-motif search were limited to 10kb around the respective transcriptional start sites by using the RcisTarget-implemented “hg19-tss-centered-10kb-7species.mc9nr.feather” motifRanking file. Prediction was performed using the cisTarget function and the resulting top 3 predicted TF, according to their normalized enrichment scores (NES), were selected for each heatmap cluster.

#### Gene set ontology enrichment analysis

Gene set ontology enrichment analysis using the heatmap clusters as input was performed on the gene sets from the Gene Ontology (GO) biological process (BP) database (60, 61) and the Hallmark gene sets (62) using the R package clusterProfiler (version 4.0.5) (63). Ontologies with the highest and statistically significant enrichment were used for presentation.

#### Gene set variation analysis

For the enrichment of the genes included in the four different clusters of the DE heatmap (PBMC data) and for the enrichment of the four different transcriptional signatures for the *in vitro* verification of drugs, the GSVA package (version 1.40.1) (64) was applied.

#### Flow analysis

After pre-processing, compensated fluorescence intensities were exported from FlowJo (BD, v. 10.7.1). Exported.fcs files were imported in R with the flowCore package (v. 2.2.0). Fluorescence intensities were auto-logicle transformed, used for dimensionality reduction using the UMAP algorithm (umap package v. 0.2.7.0) (65) and clustered using the Phenograph package (v. 0.99.1) (66). Cell types were annotated for each cluster by respective marker expression. For visualization, the proportions of main cell types were calculated and stratified by disease group.

#### ATAC-seq analysis

Reads were aligned to human hg38 reference with bowtie2 (67). Samtools (68) was used to remove adapter offset and to create bam files. Open chromatin peaks were called using MACS2 (69), blacklisted regions (hg38-blacklist.v2.bed.gz, https://sites.google.com/site/anshulkundaje/projects/blacklists), the low covered peaks were excluded, and then the peaks were annotated with gene models from TxDb.Hsapiens.UCSC.hg38.knownGene using the ChIPseeker package (applying annotatePeaks function) (70). Downstream analysis was performed with the DESeq2 (v1.26.0) package (55). Differentially accessible regions (DAR) were detected with a |FC|>1.5 and a corrected p-value > 0.05. With these standard parameters, no DAR were identified.

#### ScRNA-seq data analysis

ScRNA-seq UMI count matrices were imported to R 4.1 and gene expression data analysis was performed using the Seurat package 4.0.4 (71, 72). Cells with more than 10% mitochondrial reads and less than 200 expressed genes were excluded from the analysis and only those genes present in more than 3 cells were considered for downstream analysis. Moreover, the genes *MT-RNR1* and *MT-RNR2* were excluded. Log-normalization, scaling, and dimensionality reduction steps were performed using the Seurat implemented functions. For scaling, the number of detected transcripts per cell was regressed out to correct for heterogeneity associated with differences in sequencing depth. For dimensionality reduction, PCA was performed on the top 2,000 variable genes identified using the vst method implemented in Seurat. Subsequently, UMAP was used for two-dimensional representation of the data structure using the first 30 PCs. Cell type annotation was based on the respective clustering results combined with the expression of known marker genes. DEG by celltype were calculated for the comparison of HIV vs control with a |log2FC|>0.25, adj. p-value<0.05 and min.pct=0.1.

#### Data integration

Data integration of the PLHIV PBMCs (this study) and the acute HIV PBMC dataset (33) were integrated using the harmony algorithm (73) based on the first 15 principal components. Prior to integration, the PLHIV dataset was subsetted for major cell types present in acute HIV. Cell type annotation was based on the respective clustering results combined with the expression of known marker genes.

#### Integrated scRNA-seq monocyte analysis

The monocyte compartment was subsetted from the integrated PBMCs and subsequently normalized, scaled, and subjected to PCA calculation. For UMAP visualization, the first 10 harmony PCs were used. After clustering the integrated monocytes with the FindNeighbors and FindClusters function from Seurat, monocyte states were annotated according to the signatures described in acute HIV (33) and cluster-specific markers, separating the monocyte population into anti-viral (*TNFSF10, ISG15, IFIT2, IFIT3*), inflammatory (*IL8, IL1B, EREG*), anti-viral/inflammatory (*CCL2, CCL4*), IFI27/30^hi^ (*IFI27, IFI30*), HLA^hi^ (*HLA-DRB1, HLA-DQA1*), resting (*S100A8, S100A9, LYZ*) and non-classical (*FCGR3A, C1QA*) monocytes.

#### Confusion matrix

For each monocyte cell state, the relative proportion across the groups (HIV, control) was visualized as a fraction of samples from the respective condition contributing to the monocyte cell state stratified by dataset (PLHIV vs. acute HIV).

#### Drug prediction

To identify drugs that reverse the gene expression signature observed in the comparison HIV vs. control for bulk RNA-seq PBMCs, bulk RNA-seq CD14 monocytes, and scRNA-seq monocytes, the drug prediction databases iLINCS (http://www.ilincs.org/ilincs/), and CLUE (https://clue.io/) were accessed. As input for the drug prediction, the top 1000 (iLINCS) or the top 100 (CLUE) DEGs were used. Drugs reversing the HIV gene expression signature (defined by a negative score) comprised a total of 519 unique drugs. Using the iLINCS API (https://github.com/uc-bd2k/ilincsAPI/blob/master/usingIlincsApis.Rmd), every gene expression signature from each drug listed in the signature libraries iLINCS chemical perturbagens (LINCSCP), iLINCS targeted proteomics signatures (LINCSTP), Disease-related signatures (GDS), Connectivity Map signatures (CMAP), DrugMatrix signatures (DM), Transcriptional signatures from EBI Expression Atlas (EBI), Cancer therapeutics response signatures (CTRS), and Pharmacogenomics transcriptional signatures (PG) was downloaded. Labeling was performed in the following principle: “drug name”_”database”_”database ID”. Signatures were ordered by fold change, and only the top 300 genes were used. This resulted in a total of 17,641 unique drug signatures each with an up- and downregulated set. Subsequently, GSEA was performed on the sequencing data for every up- and down-regulated set for each drug and each cluster comparison. The resulting normalized enrichment scores (NES) were used to calculate the delta NES for each drug, defined as ΔNES = NES (down) − NES (up), ergo the difference of the NES from the downregulated set and the NES from the upregulated set of each respective drug. These ΔNES values were then k-mean clustered (k = 40). The cluster with the highest ΔNES values for both CD14 and PBMCs was chosen and uniquely present drugs were shown. The leading edge genes of the downregulation signatures of these drugs (cluster 43) were examined, and the frequency was counted (recurring target genes).

#### Data visualization

For data visualization, the R packages Seurat, ggplot2 (version 3.3.5) (74), (https://ggplot2.tidyverse.org), pheatmap (version 1.0.12), and ComplexHeatmap (version 2.8.0) (75) were used.

## Data availability statement

The datasets presented in this study can be found in online repositories. Bulk RNA-seq datasets and single-cell RNA-seq data have been deposited at the European Genome-phenome Archive (EGA) and are publicly available under the accession number EGAS00001007460.

All original code is stored on FASTGenomics: https://beta.fastgenomics.org/p/HIV_Pilot. Any additional information required to reanalyze the data reported in this paper is available from the lead contact upon request.

## Ethics statement

Ethical approval was granted by the Ethical Committee of the Radboud University Medical Center Nijmegen, the Netherlands under registration number NL68056.091.18). Written consent was obtained from all participants involved in this study and experiments were conducted according to the Declaration of Helsinki principles. The studies were conducted in accordance with the local legislation and institutional requirements. The participants provided their written informed consent to participate in this study. Written informed consent was obtained from the individual(s) for the publication of any potentially identifiable images or data included in this article.

## Author contributions

RK: Writing – original draft, Writing – review & editing, Data curation, Formal Analysis, Investigation, Visualization. LB: Data curation, Formal Analysis, Investigation, Writing – review & editing. JS: Data curation, Investigation, Validation, Writing – review & editing, Formal Analysis. SW-H: Data curation, Formal Analysis, Investigation, Writing – review & editing. MJ-C: Data curation, Investigation, Project administration, Resources, Writing – review & editing. EB: Writing – review & editing, Investigation. NR: Data curation, Formal Analysis, Investigation, Writing – review & editing. AH: Investigation, Writing – review & editing. MH: Investigation, Writing – review & editing, Formal Analysis. MN-G: Investigation, Formal Analysis, Writing – review & editing. TO: Writing – review & editing, Resources. WH: Resources, Writing – review & editing, Data curation, Investigation. LW: Resources, Writing – review & editing. AS: Resources, Writing – review & editing, Methodology. KH: Writing – review & editing, Investigation. MB: Data curation, Writing – review & editing. MDB: Resources, Supervision, Writing – review & editing. MN: Resources, Supervision, Conceptualization, Funding acquisition, Writing – review & editing. LJ: Conceptualization, Funding acquisition, Resources, Supervision, Writing – review & editing. AV: Conceptualization, Funding acquisition, Resources, Supervision, Writing – review & editing. JS: Conceptualization, Funding acquisition, Resources, Supervision, Writing – review & editing, Writing – original draft, Investigation. AA: Conceptualization, Funding acquisition, Resources, Supervision, Writing – original draft, Writing – review & editing, Project administration, Data curation, Investigation.

## Supplementary material

Supplementary Figure 1 Blood transcriptomes of PLHIV are dominated by monocyte-related proinflammatory gene programs. **(A)** Overview of age and sex of the cohort by disease group. **(A)** Multicolor flow cytometry (MCFC) cell distribution for HIV and controls. **(B)** Number of DEG for the comparison HIV vs. Ctrl in bulk PBMCs transcriptomes; IHW multiple comparison adjustment and false discovery rate (FDR) cutoff of 5%, significant fold change of >|1.5|. **(C)** Gene set variation analysis (GSVA) of the genes from the four clusters of the DE heatmap (from ).

Supplementary Figure 2 Bulk transcriptomics of monocytes in PLHIV mainly reveals IFN-signaling. **(A)** Boxplots of clinically relevant markers measured in the serum of PLHIV. References as blue bars (1). **(B)** Overview of MACS CD14 positive selection. **(C)** Number of DEG for the comparison HIV vs. Ctrl in bulk RNA-seq CD14; IHW multiple comparison adjustment and false discovery rate (FDR) cutoff of 5%, significant fold change of >|1.5|. **(D)** Boxplot and whisker of selected genes.

Supplementary Figure 3 ‘Anti-viral’ monocyte state is persistent in PLHIV. **(A)** Cell type marker expression of the PLHIV dataset for all identified cell types. **(B)** Overlap of up-regulated DEG from monocytes extracted from scRNAseq and bulk CD14^+^ transcriptomes (**Supplementary Table S3**). Genes contributing to the IFN-γ or IFN-α response pathways are indicated for the intersection as well as the uniquely identified DEG. **(C)** UMAP of integrated PBMCs from PLHIV and acute HIV split by dataset (total n= 89,500 cells, each 30,000 cells depicted). **(D)** Cell type marker expression of the integrated HIV dataset for all included cell types. **(E)** Monocyte cell state marker of the integrated monocytes from PLHIV and acute HIV. **(F)** Mapping of HIV-specific (upregulated) DEG of resting and non-classical monocyte states (for DEG see **Supplementary Table S3**) to PBMC clusters from . **(G)** Integrated monocyte UMAP subsetted for chronic HIV and stratified by donor.

Supplementary Figure 4 Drug repurposing to reverse monocyte reprogramming in PLHIV. **(A)** Enrichment of recurring target genes from cluster 43 in monocyte states of the integrated monocyte analysis (see ). **(B)** Included samples by treatment condition after quality control (QC) for the *in vitro* verification experiment. **(C)** Number of DEG (adj. p-value<0.05, |FC|>2, IHW) for each treatment vs. control (DMSO). **(D)** Heatmap showing the union of top leading edge genes of each signature (from ) for each treatment ranked by adj. p-value.

Supplementary Table 1 Donor overview.

Supplementary Table 2 Functional enrichment (GO and Hallmark) and transcription factor (TF) prediction of bulk RNA-seq PBMC heatmap clusters (related to **Figure 1**) and MCFC marker.

Supplementary Table 3 ScRNA-seq monocytes DEG (related to **Figure 3**).

Supplementary Table 4 Predicted drug clusters and signatures (related to **Figure 4**).
