# Supplementary material for: Identification of drug candidates targeting monocyte reprogramming in people living with HIV
Source: Front Immunol. 2023 Nov 20;14:1275136. doi: 10.3389/fimmu.2023.1275136 (PMC10703486; doi:10.3389/fimmu.2023.1275136)
Supplement: Supplementary Figure 1 — Blood transcriptomes of PLHIV are dominated by monocyte-related proinflammatory gene programs. (A) Overview of age and sex of the cohort by disease group. (A) Multicolor flow cytometry (MCFC) cell distribution for HIV and controls. (B) Number of DEG for the comparison HIV vs. Ctrl in bulk PBMCs transcriptomes; IHW multiple comparison adjustment and false discovery rate (FDR) cutoff of 5%, significant fold change of >|1.5|. (C) Gene set variation analysis (GSVA) of the genes from the four clusters of the DE heatmap (from ). [file DataSheet_1.pdf]

Figure S1

A

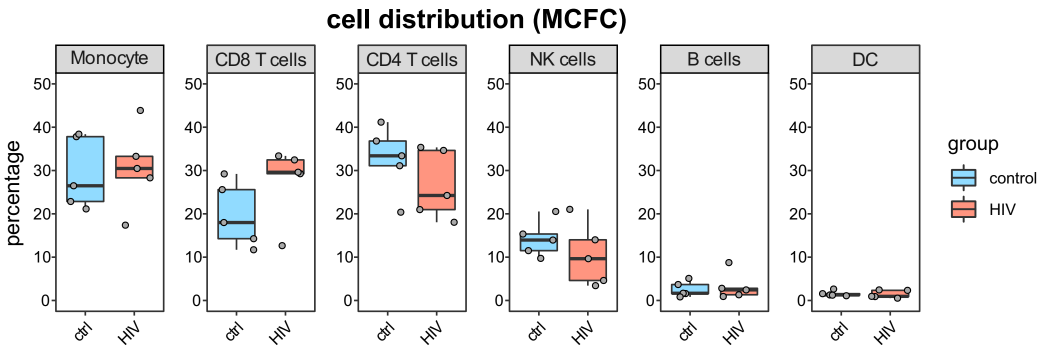

B

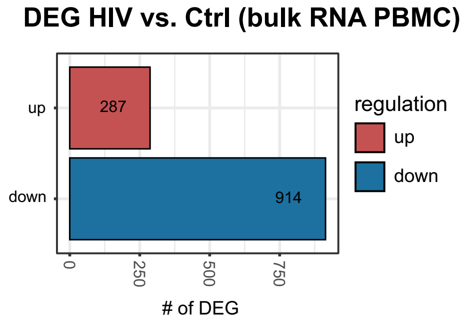

C

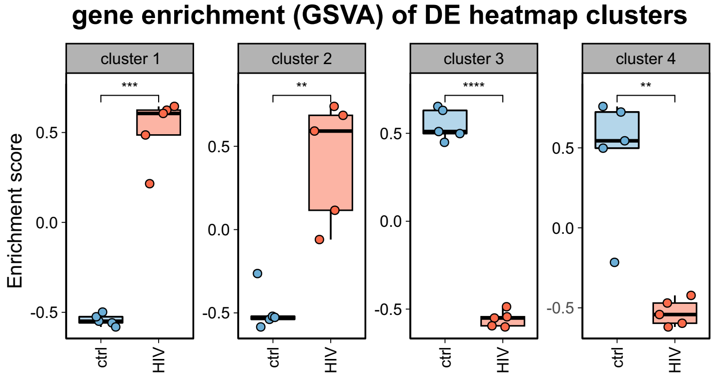

Figure S2

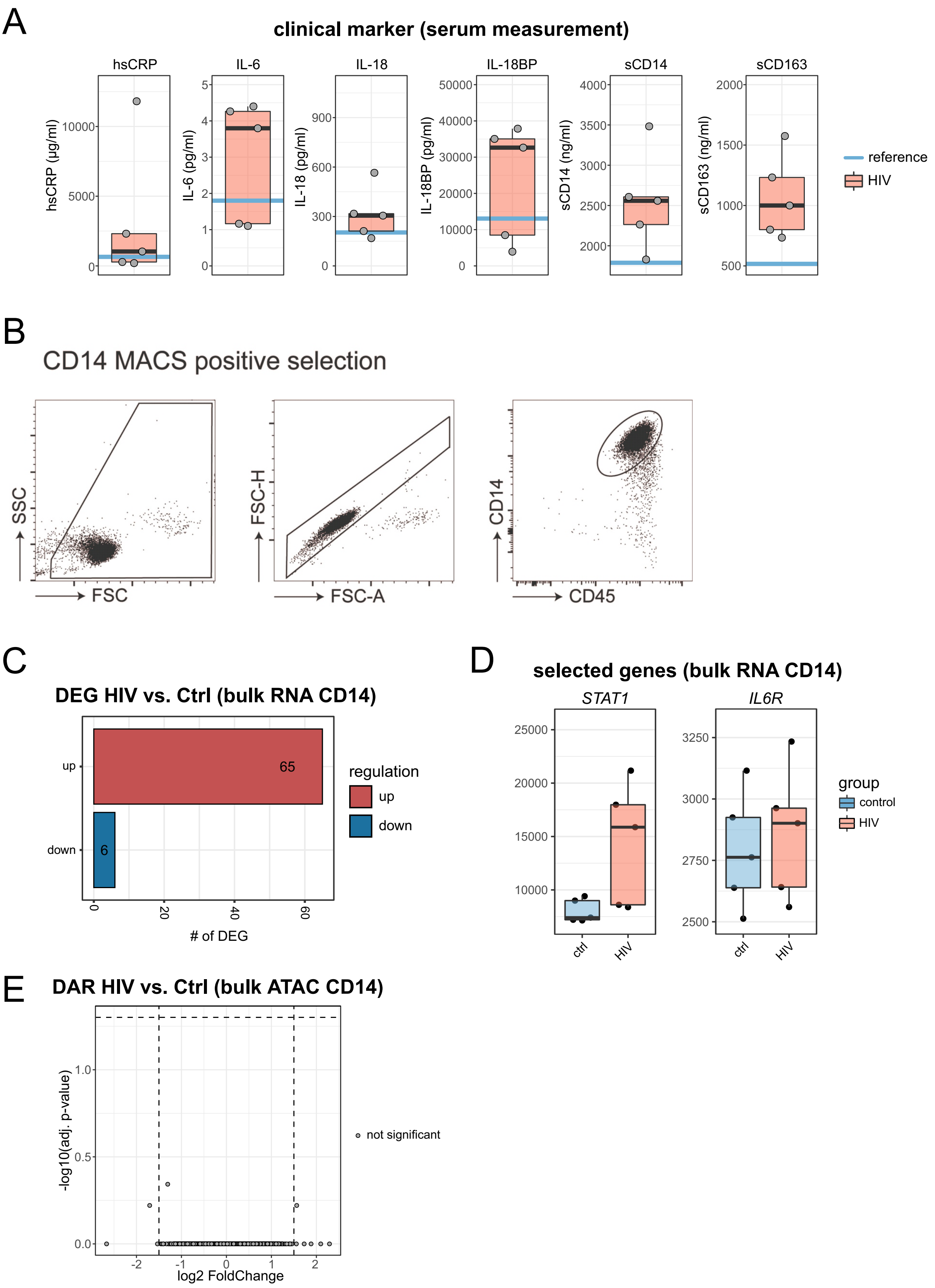
A
clinical marker (serum measurement)
hsCRP
IL-6
IL-18
IL-18BP
sCD14
sCD163
hsCRP (µg/ml)
IL-6 (pg/ml)
IL-18 (pg/ml)
IL-18BP (pg/ml)
sCD14 (ng/ml)
sCD163 (ng/ml)
reference
HIV
B
CD14 MACS positive selection
SSC
FSC
FSC-H
FSC-A
CD14
CD45
C
DEG HIV vs. Ctrl (bulk RNA CD14)
up
down
65
6
# of DEG
regulation
D
selected genes (bulk RNA CD14)
STAT1
IL6R
ctrl
HIV
group
control
E
DAR HIV vs. Ctrl (bulk ATAC CD14)
-log10(adj. p-value)
log2 FoldChange
not significant

Figure S3

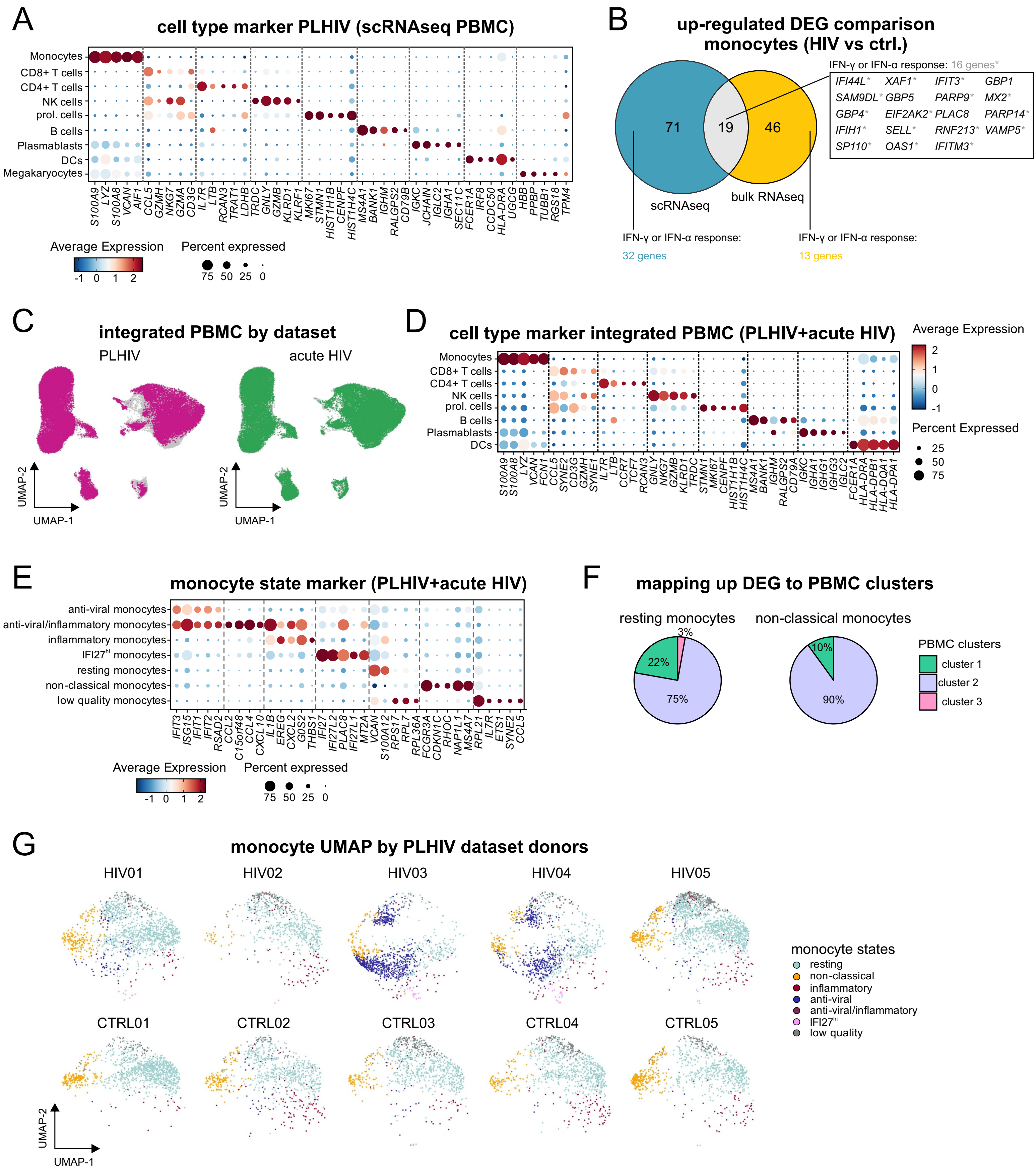
A
cell type marker PLHIV (scRNAseq PBMC)
Monocytes
CD8+ T cells
CD4+ T cells
NK cells
prol. cells
B cells
Plasmablasts
DCs
Megakaryocytes
S100A9 LYZ S100A8 VCAN AIF1 CCL5 GZMH NKG7 GZMA CD3G IL7R LTB RCAN3 TRAT1 LDHB TRDC GNLY GZMB KLRD1 KLRF1 MKI67 STMN1 HIST1H1B CENPF HIST1H4C MS4A1 BANK1 IGHM RALGPS2 CD79B IGKC JCHAIN IGLC2 IGHA1 SEC11C FCER1A IRF8 CCDC50 HLA-DRA UGCG HBB PPBP TUBB1 RGS18 TPM4
Average Expression
-1 0 1 2
Percent expressed
75 50 25 0
B
up-regulated DEG comparison monocytes (HIV vs ctrl.)
71
19
46
scRNAseq
bulk RNAseq
IFN-γ or IFN-α response: 16 genes*
IFI44L* XAF1* IFIT3* GBP1
SAM9DL* GBP5 PARP9* MX2*
GBP4* EIF2AK2* PLAC8 PARP14*
IFIH1* SELL* RNF213* VAMP5*
SP110* OAS1* IFITM3*
IFN-γ or IFN-α response:
32 genes
IFN-γ or IFN-α response:
13 genes
C
integrated PBMC by dataset
PLHIV
acute HIV
UMAP-2
UMAP-1
D
cell type marker integrated PBMC (PLHIV+acute HIV)
Monocytes
CD8+ T cells
CD4+ T cells
NK cells
prol. cells
B cells
Plasmablasts
DCs
S100A9 S100A8 LYZ VCAN FCN1 CCL5 SYNE2 CD3G GZMH SYNE1 IL7R LTB CCR7 TCF7 RCAN3 GNLY NKG7 GZMB KLRD1 TRDC STMN1 MKI67 CENPF HIST1H1B HIST1H4C MS4A1 BANK1 IGHM RALGPS2 CD79A IGKC IGHA1 IGHG1 IGHG3 IGLC2 FCER1A HLA-DRA HLA-DPB1 HLA-DQA1 HLA-DPA1
Average Expression
2 1 0 -1
Percent Expressed
25 50 75
E
monocyte state marker (PLHIV+acute HIV)
anti-viral monocytes
anti-viral/inflammatory monocytes
inflammatory monocytes
IFI27hi monocytes
resting monocytes
non-classical monocytes
low quality monocytes
IFIT3 ISG15 IFIT1 IFIT2 RSAD2 CCL2 C15orf48 CCL4 CXCL10 IL1B EREG CXCL2 G0S2 THBS1 IFI27 IFI27L2 PLAC8 IFI27L1 MT2A VCAN S100A12 RPS17 RPL7 RPL36A FCGR3A CDKN1C RHOC NAP1L1 MS4A7 RPL21 IL7R ETS1 SYNE2 CCL5
Average Expression
-1 0 1 2
Percent expressed
75 50 25 0
F
mapping up DEG to PBMC clusters
resting monocytes
non-classical monocytes
3%
22%
75%
10%
90%
PBMC clusters
cluster 1
cluster 2
cluster 3
G
monocyte UMAP by PLHIV dataset donors
HIV01 HIV02 HIV03 HIV04 HIV05
CTRL01 CTRL02 CTRL03 CTRL04 CTRL05
monocyte states
resting
non-classical
inflammatory
anti-viral
anti-viral/inflammatory
IFI27hi
low quality
UMAP-2
UMAP-1

Figure S4

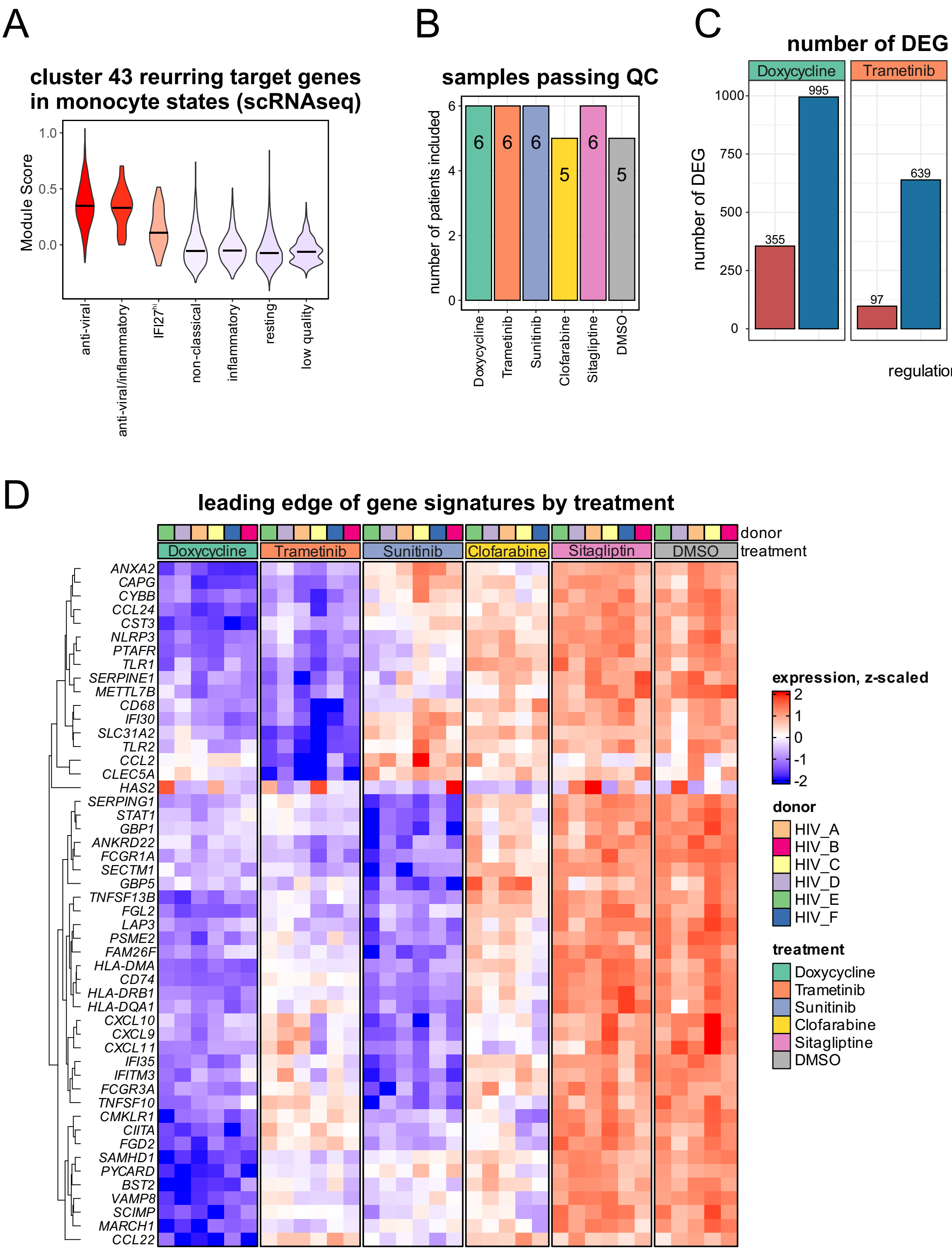
A
cluster 43 reurring target genes
in monocyte states (scRNAseq)
Module Score
1.0
0.5
0.0
anti-viral
anti-viral/inflammatory
IFI27
non-classical
inflammatory
resting
low quality
B
samples passing QC
number of patients included
6
4
2
0
Doxycycline
Trametinib
Sunitinib
Clofarabine
Sitagliptine
DMSO
C
number of DEG for treatment vs DMSO
Doxycycline
Trametinib
Sunitinib
Clofarabine
Sitagliptin
number of DEG
1000
750
500
250
0
355
995
97
639
218
421
291
201
109
105
regulation
up
down
D
leading edge of gene signatures by treatment
donor
treatment
Doxycycline
Trametinib
Sunitinib
Clofarabine
Sitagliptin
DMSO
ANXA2
CAPG
CYBB
CCL24
CST3
NLRP3
PTAFR
TLR1
SERPINE1
METTL7B
CD68
IFI30
SLC31A2
TLR2
CCL2
CLEC5A
HAS2
SERPING1
STAT1
GBP1
ANKRD22
FCGR1A
SECTM1
GBP5
TNFSF13B
FGL2
LAP3
PSME2
FAM26F
HLA-DMA
CD74
HLA-DRB1
HLA-DQA1
CXCL10
CXCL9
CXCL11
IFI35
IFITM3
FCGR3A
TNFSF10
CMKLR1
CIITA
FGD2
SAMHD1
PYCARD
BST2
VAMP8
SCIMP
MARCH1
CCL22
expression, z-scaled
2
1
0
-1
-2
donor
HIV_A
HIV_B
HIV_C
HIV_D
HIV_E
HIV_F
treatment
Doxycycline
Trametinib
Sunitinib
Clofarabine
Sitagliptine
DMSO
